# Understanding behavioural drivers of iron and folic acid supplementation and vitamin A- and iron-rich food consumption among women and adolescent girls in Niger

**DOI:** 10.1017/S1368980024002192

**Published:** 2024-11-04

**Authors:** Abigail Conrad, Jen Burns, Lisa Sherburne, Mamoudou Djibo Kaled, Hadiara Souley, Jennifer Nielsen

**Affiliations:** 1 Results for Development, Washington, DC, USA; 2 USAID Advancing Nutrition, Arlington, VA, USA; 3 Helen Keller International, Niamey, Niger; 4 John Snow International, Boston, MA, USA

**Keywords:** Micronutrient-rich foods, Iron-folic acid, Supplementation, Dietary practices, Gender norms

## Abstract

**Objective:**

This study examined drivers and barriers to iron and folic acid supplementation and consumption of iron- and vitamin A-rich foods among women of reproductive age and adolescent girls in Niger.

**Design:**

This was an exploratory qualitative study using focus group discussions and semi-structured interviews.

**Setting:**

This study was conducted in the rural areas of three districts in Maradi and Zinder regions of southern Niger.

**Participants:**

The study included a purposive sample of 56 WRA (15–49 years), 34 non-pregnant adolescent girls (10–14 years), 105 family members and community leaders, 32 health workers and 12 national and regional level stakeholders.

**Results:**

Respondents demonstrated widespread knowledge of recommendations for iron and folic acid during pregnancy and had positive attitudes towards supplements. However, supply barriers were significant, and adherence was limited by side effects, among other constraints. Despite knowledge about and positive attitudes towards micronutrient-source foods, respondents reported that limited food access was an overriding constraint to increasing consumption. Women of reproductive age were seen as nutritionally vulnerable; however, husbands were often not able to provide sufficient nutritious food, and there was a cultural expectation that wives serve themselves food last. Adolescent girls were not seen as nutritionally vulnerable, and there was little family support for their consumption of nutritious foods.

**Conclusions:**

Projects should work with local actors to develop multi-pronged solutions that address supply and access barriers for iron and folic acid and micronutrient-rich foods and to improve social support for nutrition.

Insufficient iron and vitamin A intake is a concern in Niger as the national prevalence rate for anaemia is 59 %^(1)^, and Niger has one of the highest age-standardised incidences of vitamin A deficiency (VAD) and disability-adjusted life years due to VAD in the world^(2)^.

Diets are typically poor in rural Niger, which contributes to inadequate iron and vitamin A intake among women and adolescent girls. Only 46·8 % of women of reproductive age (WRA) consume minimum acceptable dietary diversity nationally, while 25 and 57·9 % do in Maradi and Zinder, respectively^(3)^. In Zinder, dietary intake of iron was inadequate for 47 % of pregnant women, and 52 % of pregnant women consumed inadequate amounts of vitamin A^(4)^. The literature identifies several factors contributing to this limited dietary diversity in Niger. Insufficient availability and affordability of diverse, nutrient-rich foods are caused by challenging agro-ecological conditions, underdeveloped market systems, shocks and climate change; households’ low purchasing power; women’s low status and decision-making power and high labour burden; and certain cultural and social norms^(5)^. However, the current evidence base does not address drivers and barriers that affect women’s and adolescent girls’ consumption of vitamin A- and iron-rich foods specifically. This evidence gap is particularly pronounced for adolescent girls in Niger, who had the world’s highest adolescent fertility rate in 2021 (170 births per 1000 women ages 15–19)^(6)^.

Iron and folic acid (IFA) supplementation during pregnancy is recommended to improve pregnancy outcomes and prevent maternal anaemia^(7)^. However, as of the most recent data from 2012, only 29 % of pregnant women^(1)^ took the recommended 90 d course of IFA (60 milligrams of elemental iron and 400 micrograms of folic acid)^(1,8)^. IFA supplementation is provided through antenatal care (ANC); however, the provision of IFA is not consistent at ANC, and adherence remains an issue due to side effects and acceptability^(9,10)^. Studies in Niger have focused on ANC delivery, with few exploring community-based solutions.

The [project name removed for anonymity] (2018–2024), a global multi-sectoral nutrition project, conducted this study to understand drivers and barriers for two behaviours to inform project recommendations:

- IFA supplementation among pregnant women;
- Consumption of iron- and vitamin A-rich foods among WRA and adolescent girls.

## Methods

### Study design

This was an exploratory qualitative study. We used focus group discussions (FGD) and semi-structured interviews. These included cognitive mapping and participatory techniques, which are particularly useful for data collection with adolescents^(11)^. The COM-B model, which stands for Capability (C), Opportunity (O), Motivation (M) and Behaviour (B), guided lines of inquiry. These three essential conditions at the centre of the model guide exploration of the barriers and drivers that need to be in place for people to practice priority behaviours^(12)^.

### Study setting

The study was conducted in three districts in southern Niger – Guidan Roumdji District in the Maradi region and Magaria District and Damagaram Takaya District in the Zinder region. These three districts were in the implementation areas for the US Agency for International Development (USAID) Resilience in the Sahel Enhanced (RISE) II initiative (2019–2025), which aims to build resilience to recurrent shocks, including economic and health shocks. This study informed the design of new nutrition interventions to improve iron and vitamin A intake to complement existing interventions to improve access to quality nutrition services and nutritious, safe foods. All three districts are largely rural and have distinct agro-ecological zones – Guidan Roumdji is in the millet and sorghum agricultural zone, Magaria is in the southern zone of market gardens and Damagaram Takaya is in the agro-pastoral zone^(13)^. We collected data in January 2022, just after the main harvest. The rainy season is approximately June to September and coincides with the lean season for agricultural communities^(14)^. As of 2012, 38·2 % of the population in Maradi and 47·2 % in Zinder lived in the lowest two wealth quintiles. The average household size in rural Niger was 5·9 and 52 % of women in Maradi, and 31 % of women in Zinder were in polygamous unions^(1)^.

### Sampling and methods

We worked with USAID RISE II and health officials to identify two communities per district within the USAID RISE II implementation areas. Within each district, we selected one community that was less than 5 km from a health centre and one that was more than 5 km away from a health centre. We also aimed for one of these communities to be primarily agro-pastoral and one to be primarily agricultural. We were not able to select one agro-pastoral in each district, however. Security was considered when selecting communities for data collection.

The study included six categories of respondents: (1) pregnant women or women (15–49 years) with a child under 5 years of age (hereafter referred to as WRA) in USAID RISE II participant households; (2) non-pregnant, in school or out of school adolescent girls (10–14 years) in USAID RISE II participant households; (3) husband or mother-in-law of WRA in USAID RISE II participant households (hereafter referred to as family members); (4) community influencers (e.g. village chiefs, presidents of women’s community groups); (5) health workers and community health volunteers; and (6) stakeholders (Ministry of Public Health, UNICEF, World Food Programme and USAID implementing partners). For each respondent group, the target sample size was estimated based on ranges from a systematic review, which found that 4–8 FGD per respondent category and 9–17 interviews are sufficient to reach data saturation for key concepts among relatively homogeneous populations^(15)^.

We used purposive sampling. In each selected community, we worked with the USAID RISE II implementing partner or the mother’s or farmer’s group leader working with USAID RISE II to select WRA, family members and adolescent girls from USAID RISE II participant households reflecting the socio-economic diversity of USAID RISE II participants in the community. They also helped identify respected influencers in the community who provided advice to families on health and nutrition or who had engaged with USAID RISE II. Health workers at the district level were selected who oversaw or procured IFA supplementation, and health workers and community health workers from the nearest health post or integrated health centre were selected who were involved in ANC. We conducted twenty-seven FGD and thirty-four interviews (individual or group) with a total sample size of 239 (Table 1).

**Table 1.** Data collection methods and numbers of respondents by geography

Data collection method	Respondent group	Total FGD/interview	Geographic level				
			National level	Damagaram Takaya District	Magaria District	Guidan Roumdji District	Total
Focus group discussions (FGD)	WRA	6 (2 per district)	N/A	23	21	12	56
	Adolescent girls 10–14 years	9 (1 per district)	N/A	9	7	6	22
	Family members	6 (2 per district)	N/A	12	20	12	44
	Community leaders	6 (2 per district)	N/A	22	24	15	61
Group interviews	Health workers	8 (1–4 per district)	N/A	3	8	13	24
	Adolescent girls 10–14 years	6 (2 per district)	N/A	4	4	4	12
Individual interviews	Health workers	8 (2–4 per district)	N/A	4	2	2	8
	Stakeholders	12 (6 national level and 3 per region)	6	3^*^		3	12
**Total**		**61**	**6**	**80**	**86**	**89**	**239**

WRA, women of reproductive age.

* Three regional level interviews in Zinder covered both Damagaram Takaya and Magaria Districts and are counted in the total under Damagaram Takaya District.

FGD were conducted separately with WRA, adolescent girls, family members and community leaders, with each FGD having 6–12 respondents. The FGD included a mix of semi-structured questions, cognitive mapping using pile sorts and participatory techniques. In the pile sort activities, we included foods that contribute to meeting these micronutrient requirements in addition to those considered micronutrient-rich (see online supplementary material, Supplemental Table S1) given the food insecurity challenges in Niger.

In addition to FGD with adolescent girls, we also used the ‘best friend’ interview approach (12). These were group interviews with two adolescent girls who were friends using an interview guide similar to the FGD guide.

Interviews with health workers (individual and small group interviews) and stakeholders (individual interviews) were semi-structured interviews with no cognitive mapping or participatory exercises.

### Data collection procedures

[Project name] contracted a firm in Niger, Conception, Études, Sécurité, Alimentaire, Formation (CESAF), to complete data collection activities, except for stakeholder interviews, which [project name] staff conducted. Data collectors participated in a multi-day training led by CESAF and overseen by [project name]. Data were collected in Hausa, Kanuri or French as preferred by respondents. [project name] developed the guides in English and translated them into French. CESAF then translated and back-translated the guides into Hausa. The guides were translated verbally by data collectors into Kanuri when applicable as Kanuri was spoken in a minority of study communities. The guides were finalised after pretesting in a community outside of the sample.

The FGD and interviews were audio recorded with consent, and the data collection firm produced detailed notes in French for coding and analysis. Structured forms were used to document the data from the cognitive mapping and participatory exercises. Direct quotations in the manuscript were transcribed from the audio recordings.

### Data analysis

We used applied thematic analysis to identify the themes and patterns in the narrative data^(16)^ and used ATLAS.ti to organise and code the narrative data. We developed a codebook with a mix of deductive and inductive codes while coding a small sample of documents. Once the codebook was finalised, two team members coded all documents. In the analysis, we disaggregated the findings by district and respondent group.

We used Excel to analyse the constrained pile sort data. For the unconstrained pile sorts, which resulted in 216 piles, we used similarity matrices, non-metric multidimensional scaling and t-distributed stochastic neighbour embedding in Python to visualise and qualitatively analyse the pile sort data (see online supplementary material, Supplemental Figs. S2 and S3).

We shared the preliminary findings with USAID implementing partners and the Ministry of Public Health through a participatory validation workshop to further refine and finalise the findings and project recommendations.

### Research ethics

Research ethics, confidentiality, data security procedures and COVID-19 precautions were covered in the data collector training. We obtained informed consent from respondents over the age of 18. For all unmarried respondents under the age of 18 (i.e. minors), we first obtained informed consent from their parents or guardians before data collection. We then obtained the adolescents’ assent to participate in the research.

## Results

We first present the respondent characteristics. Next, we present overarching social and gender norms related to food and nutrition. Then, for each behaviour – IFA supplementation and vitamin A- and iron-source food consumption – we share findings related to local knowledge and perceptions, drivers and barriers.

### Respondent and sample characteristics

Across districts, 136 respondents were female, and 91 respondents were male. Thirty-four respondents were adolescent girls aged 10–14 years, 56 were WRA (15–49 years) and 137 respondents were adults 18 years of age or older (Table 2).

**Table 2.** Respondent characteristics by district^*^

Characteristic	Damagaram Takaya	Guidan Roumdji	Magaria	Total
Gender				
Female	52	38	46	136
Male	25	26	40	91
Age (years)				
10–14	13	10	11	34
15–49	23	12	21	56
>18	41	42	54	137

* Excluding stakeholder interviews (*n* 12).

### Social and gender norms

Community perceptions about the role of nutrition in health influence social norms, including meaning and values, related to food and nutrition. Community members perceived good nutrition as having less disease, an attractive appearance for adolescent girls, weight gain for women, a shiny and radiant body and smooth skin. They also believed that good nutrition should lead to pregnant women having a safer childbirth and a healthier baby. Respondents thought that few community members fit this profile, however, as many were considered too thin. For example, one woman said:

‘Through a person who has had a good diet, you see that they are fat and chubby and their body shines. Here in our group, there is no such person. Everybody is skinny.’ (WRA, Magaria District)

Gender norms strongly shape the roles that family members play related to food and can influence women’s use of nutrition services. When asked about the characteristics of an ideal man, WRA and family members identified his ability to take care of his family’s needs, be patient, keep peace with his wife and not show flaws in public. WRA and family members expected women to be considerate, patient, discreet and respectful of their husbands, in-laws and other family members. The ideal wife also plays a supportive role in caring and sacrificing for her family and preparing and serving food to the family.

Men were the primary decision-makers about what food to purchase and what crops to grow according to respondents across groups. Women helped men with agricultural production, and respondents said that women sometimes contributed to decision-making about what crops to produce. Husbands were also responsible for purchasing IFA supplements if they were not available from the health centre.

### Iron and folic acid supplementation

#### Knowledge and perceptions of iron and folic acid supplementation and anaemia

Local knowledge and perceptions about IFA and anaemia can influence the provision and utilisation of IFA supplementation. Most women, family members and community leaders knew that women should take IFA during pregnancy and that they could obtain these supplements at the health facility during the first trimester of pregnancy. WRA, family members, health workers and community leaders knew that anaemia is common, especially in pregnant women. A woman shared:

‘Anemia is very common in our community. For example, on average, we can count more than 6 out of 10 women who are confronted with the problem of anemia during their pregnancies and childbirth.’ (WRA, Magaria District)

Respondents also saw anaemia as a serious condition, as described by one woman:

‘We equate anemia with suffering because your body expends more energy than your diet provides, so it goes without saying that your blood runs out.’ (WRA, Guidan Roumdji District)

Ministry of Public Health staff acknowledged that community members had a good understanding of anaemia. However, stakeholders noted that a lack of recent anaemia prevalence data and data on the aetiology of anaemia in the country hampers their decision-making about IFA supplementation.

#### Potential driver for iron and folic acid use

We used a vignette to explore the idea of using an ‘adherence partner’ – a family member or close friend – to support women to help improve IFA adherence^(17)^. Respondents generally reacted favourably to this idea and thought the adherence partner could support pregnant women to obtain the supplements, encourage them to take the supplements despite side effects and remind them to take the supplements daily. They identified husbands, co-wives or elder family members as potential adherence partners. Some women noted that they could share the positive experiences they had taking IFA to help motivate others and help shift norms about adherence. One woman explained:

‘The advice we have to give to women is that when you consume the IFA you will not have any problem of lack of blood during the delivery. We have consumed the IFA. We have not been disappointed, and when a woman neglects these tablets, we will tell her that all the rumors around iron are unfounded. I myself have used it, and I have been satisfied, so do not neglect the iron tablets. They are good for your health and that of your fetus.’ (WRA, Guidan Roumdji District)

#### Barriers to iron and folic acid use

Four main barriers limited IFA use – supply, distance to facilities, side effects and insufficient counselling. First, IFA supply issues were reported across respondent groups. IFA is included on Niger’s essential medicines list and should be budgeted for and procured by health facilities, according to respondents. However, stakeholders reported that IFA supply is a significant issue. UNICEF and nongovernmental organisations to a lesser extent only provide support for the procurement of IFA in some areas, according to stakeholders.

In some districts, health workers noted a lack of storage facilities and delays in transporting supplies from district stores to health centres. Many health workers also recognised a lack of systematisation of roles and responsibilities between different districts and facilities to track and order IFA supply, which could limit coordination.

This inconsistent supply meant that many women who attended ANC found that IFA was unavailable. This was commonly reported by health workers, family members and WRA. When the health centre was out of IFA, health workers instructed women to purchase IFA at pharmacies or to consume iron-rich foods like cowpeas, moringa and liver. One health worked said:

‘For us, [IFA] supplies have to be continuous. Supplies aren’t continuous. We often run out and that’s what we would like to avoid over time.’ (Health worker, Damagaram Takaya District)

Second, family members and WRA said the distance to health centres for ANC was a barrier. When pregnant, women did not risk riding a motorcycle and had to walk long distances for care. Some family members said that there are also times when health facilities turned women away because they were overcrowded or staff were unavailable.

Third, in both Maradi and Zinder, WRA and family member respondents acknowledged that even when women received IFA, they often failed to consume the recommended dose. WRA cited side effects such as vomiting or nausea, dizziness and muscle and joint pain as reasons for skipping or stopping IFA altogether. Some voiced concern that the supplements would cause excessive blood loss at delivery.

Fourth, health workers reported that they cannot spend time providing quality counselling on IFA due to a large number of clients even though women did not adhere to daily use. Many stakeholders acknowledged that counselling was non-existent due to a lack of national training protocol and counselling materials. Implementing partners also mentioned the need for supportive supervision at both the clinic and community level.

### Vitamin A- and iron-rich food consumption

#### Knowledge and perceptions of vitamin A- and iron-rich foods

Knowledge and perceptions of nutritious foods can influence dietary intake, and improving knowledge is often a focus of nutrition services and interventions. In pile sort exercises, WRA and adolescent girls correctly identified a range of foods that they saw as sources of vitamin A and iron and as foods that are good for them (Table 3).

**Table 3.** Foods that women and adolescent girls thought were good for them and a source of the relevant micronutrient, categorised by those identified as sources of vitamin A and iron

Respondent group	Sources of vitamin A	Sources of iron
Reported by both WRA and adolescent girls	Amaranth leaves^†^ Baobab leaves^†^ Liver^*^ Moringa leaves^*^ Oil (assuming fortified)^*^ Sorrel^†^ Sweet potatoes (orange flesh)^†^	Cowpeas Eggs Fish^*^ Liver^*^ Meat Moringa leaves^*^ Potatoes Soyabeans^*^ Taro^†^
Reported only by WRA	Cheese Milk^*^	Locust^†^ Tiger nut^†^
Reported only by adolescent girls	Cantaloupe^†^ Carrots^†^ Dried tomatoes Locusts^†^ Mango^*,†^ Okra^*^ Papaya^*,†^	Kilishi (dried meat) Millet

WRA, women of reproductive age.

* Respondents reported food is good for WRA.

† Respondents reported food is good for adolescent girls.

In another pile sort exercise, respondents created piles based on which foods they thought were similar, and they provided explanations for these piles. The health and nutritional properties of foods were commonly discussed (see supplementary materials for details). Preventing anaemia was the second most common health benefit identified for foods. Respondents saw vitamin A- and iron-source foods as providing a range of health benefits (Table 3).

#### Drivers for vitamin A- and iron-rich foods consumption

We identified four key drivers of vitamin A- and iron-rich food consumption – knowledge, acceptability, accessibility and social norms.

First, WRA and adolescent girls correctly identified one or more foods as sources of vitamin A and iron in nearly all focus groups (Table 3). WRA reported nutrition counselling as a source of this information.

Second, respondents largely saw these foods as good for them (Tables 3 and 4), were generally motivated to consume nutritious foods and found them to be acceptable in the local cuisine (see supplementary materials for detail). A WRA described the importance of consuming iron-source foods:

‘The foods suggested for pregnant women are given to them because they prevent anemia and improve the physical appearance of the woman’s body. She becomes bright and beautiful to look at. Those given to pregnant women strengthen the health of the mother and the child. They also promote the growth of children.’ (WRA, Magaria District)

**Table 4.** Food perceived as a source of vitamin A and iron by health or nutrition property

Health or nutrition property	Reported as source of vitamin A	Reported as source of iron
Increasing fat (*kara maski*)	None	Tiger nut
Fights hunger (*yana fidda gniwa*)	Liver Orange flesh sweet potatoes	Cowpeas Eggs Fish Kilishi (dried meat) Liver Meat Millet
Gives energy	Liver Orange flesh sweet potatoes	Liver Meat Taro
Gives strength (*karhi*) or builds the body	Cheese Liver Milk Moringa leaves	Eggs Liver Moringa leaves
Good for growth (*kayan gina jiki*)	Cantaloupe Carrots Dried tomatoes Mango Papaya	None
Has vitamins (*maganin gniwa*)	Carrots Dried tomatoes	Cowpeas Tiger nut
Improves vision	Amaranth leaves Cantaloupe Moringa leaves Papaya Sorrel	Taro Moringa leaves
Increases blood (*yana kara gini*) or fights against anaemia (*kara jini*)	Papaya Mango Cantaloupe Carrots	None
Increases vigour (*kara karfin jiki*) or liveliness (*hamzari*)	Cheese Milk Orange flesh sweet potatoes	Cowpeas Potatoes Taro
Nourishes skin (*yana djara fata*)	Amaranth leaves Cantaloupe Guava Liver Mango Oil Orange flesh sweet potatoes Papaya	Kilishi (dried meat) Liver Tiger nut

Third, respondents reported that some vitamin A- and iron-source foods were accessible. Baobab leaves, sorrel and cantaloupe were vitamin A-source foods available from their own production, and milk was available for purchase. Millet and cowpeas were iron-source foods available from their own production, and soyabeans were commonly purchased. Women and adolescent girls also reported being able to access wild foods directly, like dark leafy green vegetables (e.g. *Leptadenia hastata*, *gui*) and fruits, as they are responsible for gathering these foods.

Fourth, social norms supported husbands providing nutritious food to women. Respondents across groups explained the importance of consuming nutritious foods for the health of women and their children, and husbands were responsible for providing these foods. Community leaders said they encouraged husbands to provide their pregnant wives with vitamin A- and iron-source foods.

#### Barriers to consumption of vitamin A- and iron-rich foods

Two key barriers constrained the consumption of vitamin A- and iron-rich foods – limited access and social and gender norms.

First, while some vitamin A and iron-source foods were accessible as discussed above, many others were not commonly available or were seen as unaffordable. Household agricultural production was an important source of food for respondents, but households typically ran out of their stored harvests well before the next rainy season. A community leader in Magaria described quickly running out of stores from the harvest, which is from October to December:

‘Even millet, which is our staple food and produced locally, is often lacking because the production is generally consumed by May. This year, it will not even cover the month of April.’ (Community leader, Magaria District)

Respondents in Damagaram Takaya District and Magaria District reported having to rely on market purchases more than respondents in Guidan Roumdji. Adolescent girls also explained that the monotony of their diet and being served dishes that did not taste good reduced the quantity they consumed.

Second, social and gender norms modified the effect of food insecurity on women’s and adolescent girls’ consumption in several ways. Respondents consistently reported that women and adolescent girls were largely restricted to consuming what was available in the home, which women and adolescent girls had little control over. Adolescent girls were not involved in making decisions about what crops the family grew, and only a few said they would request specific foods from their fathers.

Intrahousehold food allocation norms deprioritized women and adolescent girls, which constrained their consumption in a context of scarcity. Women and adolescent girls had limited entitlement to food as they were typically expected to prioritise food for other family members, including children, over themselves. Adolescent girls were typically served second to last, and the wife of the household head served herself last. When there is insufficient food for everyone in the household, respondents reported that women may not be served enough food, and a few adolescent girls reported skipping meals. For example:

‘Women prepare [food] and serve other family members before themselves, and they are proud to do so, even if they receive less food than other family members.’ (Family member, Magaria District)

Power relations within the household reinforce these practices. The wife serves her husband’s sisters before serving herself, even if she is pregnant. Some female respondents noted that their mothers- and sisters-in-law insisted on enforcing the norms that the husband’s family comes first in the distribution of food.

Three, despite support for women’s nutrition, social support for adolescent girls’ consumption of a healthy diet was limited. Communities did not see adolescent girls as having unique nutritional needs or as nutritionally vulnerable. Further, some family members described the father’s responsibility to provide for unmarried adolescent girls as a ‘burden’ and that they were not entitled to certain types of rich foods.

## Discussion

Nutrition services often focus on providing supplies and training health providers to change behaviour by delivering technically accurate recommendations about nutrition, rather than addressing complex behavioural drivers^(18)^. Our findings demonstrate the complexity of behavioural drivers affecting iron and vitamin A intake. As is outlined in the UNICEF Conceptual Framework on the Determinants of Maternal and Child Nutrition, resources and norms are key enabling determinants of healthy diets and adequate nutrition services^(19)^. WRA, their family members and community influencers in rural Niger demonstrated knowledge about the benefits of IFA supplementation, a diverse diet for women and vitamin A- and iron-rich foods that were acceptable in the local cuisine. Yet few women complete the recommended course of IFA supplements, and even fewer women consume a diverse diet. Respondents recognised barriers other than a lack of knowledge for inadequate intake, namely, IFA and food access barriers and unequal social and gender norms. These enabling determinants provide the necessary preconditions for women to access and have social support to utilise IFA and vitamin A- and iron-rich foods.

### Iron and folic acid supplementation

Prior research on IFA supplementation in Niger and elsewhere has shown that poor ANC attendance and inconsistent provision of IFA at ANC due to supply chain constraints lowers IFA adherence^(9,20–22)^. Health workers and stakeholders recommended engaging community health workers to reduce the distance and time constraints, which is consistent with previous research^(20)^. Our study confirms previous findings in Niger and other countries that negative side effects also lower IFA adherence and counselling is inadequate to help women manage side effects^(20–25)^. National-level policies and budgets should ensure quality training and consistent IFA supply.

Our study identifies a promising solution to improve IFA adherence in this context, previously tried in Eastern Africa^(17)^. WRA and community respondents identified family and community support as a key enabler for accessing and adhering to IFA supplements. Due to gender roles, women are dependent on husbands and family members for financial resources and agreement to take time away from tasks when seeking services. They were open to the idea of selecting someone as an ‘adherence partner.’ Women felt that this would motivate them to take supplements by feeling more supported and cared for and believed that husbands, co-wives or mothers-in-law could be helpful to access and take the supplements. Thus, as the health system works to improve supply and accessibility, projects and services could increase demand and adherence by using a family systems approach to introduce the concept of an adherence partner. The potential effectiveness of increasing social support is bolstered by evidence from Niger showing that women are more likely to attend ANC and adhere to IFA if they receive advice about it from their husbands^(11)^.

### Vitamin A- and iron-rich food consumption

Previous research has shown that chronic food insecurity and the high cost of a nutritious diet limit the quality and diversity of diets in Niger^(5,26–28)^. Dietary assessments show that women’s dietary diversity was low in Maradi and Zinder^(4,29)^ and that iron and vitamin A intake was inadequate for approximately half of pregnant women in Zinder^(4)^. Our study builds on this evidence by identifying drivers and barriers to the consumption of vitamin A- and iron-rich foods specifically. We found that limited food access was an overriding constraint to increasing consumption. In this context of food insecurity, social and gender norms that limit access to diverse foods are critically important and are increasingly recognised as an important influence on nutrition^(30)^. While cultural and religious tenets that expected men to provide food and care for their families were supportive of nutrition, women were largely dependent on male decision-making and income to access food. Women were expected to serve themselves food last at mealtimes and to sacrifice for other family members. In communities and households with limited access to food, this can lead to women eating less than others. This aligns with Lentz’s concept of ‘burdened agency’ in which women actively make decisions around food; however, they make these decisions in a context of power inequities, and their decisions can contribute to their own undernutrition^(31)^. These norms, which are internalised by women and externally sanctioned and reinforced by mothers-in-law and other family members, coupled with little control over resources, produce a context in which women have little and indeed have burdened agency. Our findings align with work on gender inequality^(32)^ and its contribution to greater food insecurity and nutritional vulnerability in Niger^(33–35)^. Our findings also align with existing literature showing that socio-cultural norms determine intrahousehold food allocation, which results in inadequate consumption in contexts of scarcity for those with lower status or perceived need^(36–38)^.

Our study helps fill a literature gap on adolescent girls’ diets in Niger amid growing recognition of the importance of nutrition in adolescence and during adolescent pregnancy^(39,40)^. We found that respondents did not see adolescent girls as nutritionally vulnerable, and some male heads of households saw feeding them as a burden. Adolescent girls in rural Niger had low social standing in the household and received little priority. Their food access was constrained, as it was largely limited to what was available in their households and, to a lesser extent, what was available in their local food environments, including foods they could gather from the wild. Our findings are consistent with research in other countries showing that dependence on household food access, unequal gender norms and limited agency constrain the consumption of nutritious foods among adolescent girls^(40,41)^.

Addressing inadequate intake of vitamin A- and iron-rich foods requires a dual approach to improve both household access to nutritious foods and women’s and adolescent girls’ access to that food within the household. In Niger, and as is needed globally^(42)^, systems-level reforms are needed to strengthen the resilience and equity of agrifood systems and the availability of nutritious foods in local food environments. A promising avenue may be to support women to increase production of low-input, micronutrient-rich crops they already grow in their gardens, such as baobab, sorrel, moringa and indigenous dark leafy green vegetables and fruits. Beyond addressing household food access, projects should include social and behaviour change activities at the community level to enhance social and family support for women’s and adolescent girls’ consumption of micronutrient-rich foods.

### Limitations

The study was limited by the small, purposive sample size. In addition, the study was conducted in USAID RISE II implementation areas, so respondents were exposed to interventions that aimed to improve access to quality nutrition services and to increase access to and consumption of safe, nutritious foods. Respondents’ experiences and perceptions may differ from communities not receiving these interventions, such as having more positive attitudes about nutrition due to project exposure. In addition, there may have been response bias related to social desirability or to encourage more project support. Two translation limitations were that the guides were translated verbally into Kanuri and the recordings in Hausa and Kanuri were transcribed into French before analysis. Future studies should include pregnant adolescent girls and disaggregate 15–19-year-olds from older WRA as a sizable nutritionally and socially vulnerable group in Niger. Mixed methods studies would also help build the evidence base by allowing for an assessment of dietary intake and socio-economic status along with qualitative data on practices and perceptions. Qualitative data collection in future studies could be strengthened by using participatory observation to further explore practices and the influence of social and gender norms.

## Conclusion

This study in rural Niger emphasises the need to examine and address systems- and community-level drivers and barriers to improving IFA adherence and consumption of micronutrient-rich foods among women and adolescent girls. It also highlights the importance of examining socio-cultural norms and power relations when seeking to understand nutrition-related practices, whether rooted in health or food systems. Donor-funded projects should work with local actors to develop multi-pronged solutions that will address supply and access constraints in addition to improving social and family support for nutrition.

## Supporting information

Conrad et al. supplementary material Conrad et al. supplementary material

## Supplementary material

For supplementary material accompanying this paper, visit https://doi.org/10.1017/S1368980024002192
