# Supplementary material for: Understanding behavioural drivers of iron and folic acid supplementation and vitamin A- and iron-rich food consumption among women and adolescent girls in Niger
Source: Public Health Nutr. 2024 Nov 4;27(1):e249. doi: 10.1017/S1368980024002192 (PMC13740147; doi:10.1017/S1368980024002192)
Supplement: Supplementary file 1 [file S1368980024002192sup001.docx]

# Supplementary Materials

## Pile Sort Analysis

The pile sorts were done using 47 food cards from a food list developed by USAID Advancing Nutrition and USAID RISE II staff to include common foods consumed in the districts and locally available foods that are sources of iron and vitamin A (Table S1). One pile sort activity was an unconstrained pile sort in which respondents created any number of piles based on which foods go together. The second pile sort activity was a constrained pile sort in which respondents were asked to create a pile of foods with iron and of foods with vitamin A.

For the unconstrained pile sorts, we first created an aggregate similarity matrix and used metric MDS to generate an aggregate proximity matrix^(1)^. However, the MDS had a stress of 0.35, which indicates a poor goodness of fit^(2)^ and visual inspection of the resulting plot did not show any strong patterns Although MDS is the classical method for pile sort analysis, modern non-linear methods for visualizing high-dimensional data provide superior performance on complex, real-world data; we applied t-SNE, which is widely used in diverse application areas^(3)^. The t-SNE visualization of the entire dataset somewhat improves over MDS, but the analysis appeared limited by the design of the exercise. Specifically, the pile sort exercise was unconstrained, which meant that respondents created piles based on different types of criteria. Since the respondents provided descriptions of the piles they created, we identified categories for these descriptions. The two dominant categories were eat/cooking (n=101 piles) (e.g., food groups, foods that are cooked together) and health/nutrition (n=96 piles) (e.g., foods that give energy) (Figure S2). The t-SNE visualization for these two subsets (Figure S1 and Figure S2, respectively) showed useful clusters, although some outliers are still present, and these results were used in the findings.

**Table S1.** Locally Available Foods Used for Pile Sort Exercises and Whether They are a Source of Iron or Vitamin A

| **English Name** | **Hausa Name** | **Iron Source Food** | **Vitamin A Source Food** |
| --- | --- | --- | --- |
| Rice | *Shinkafa* |  |  |
| Oil | *Mai* |  | X |
| Wheat | *Alkama* | X |  |
| Cassava | *Rogo* |  |  |
| Peanuts | *Gujia* | X |  |
| Baobab leaves | *Miya kuka* | X | X |
| Eggs | *Koye* | X | X |
| Soybeans | *Waken awara* | X |  |
| Cowpea | *Wake* | X |  |
| Liver | *Anta* | X | X |
| Fish | *Kihi* | X | X |
| Milk | *Nono* |  | X |
| Cheese | *Tchuku* |  | X |
| Locusts | *Fara* | X | X |
| Moringa leaves | *El makka* | X | X |
| Sorrel | *Yakua* | X | X |
| Baobab fruit | *Kuka* | X |  |
| Millet | *Hatsi* | X |  |
| Guinea corn | *Ja-dawa* | X |  |
| Sorghum | *Fara-dawa* | X |  |
| Carrots | *Karoti* |  | X |
| Cabbage | *Kabeji* |  |  |
| Sweet potatoes (orange flesh) | *Dankali* |  | X |
| Dried tomatoes | *Toumati boussasa* | X | X |
| Mango | *Mangoro* |  | X |
| Papaya | *Godda* |  | X |
| Orange | *Lemu* |  |  |
| Amaranth leaves | *Dangnan haki* | X | X |
| Yam | *Doya* |  |  |
| Cantaloupe | *Malo* |  | X |
| Banana | *Banana* |  |  |
| Eggplant | *Yalon Tourawa* |  |  |
| Taro | *Gwaza/mankani* | X |  |
| Wild eggplant | *Yalo* |  |  |
| Pumpkin | *Kabewa* |  | X |
| Tiger nut | *Aya* | X |  |
| Okra | *Kubewa* |  | X |
| Meat | *Nama* | X |  |
| Potatoes | *Dankalin turawa* | X |  |
| Onions | *Albassa* |  |  |
| Soybean curds | *Awara* | X |  |
| Jerky | *Kilishi* | X |  |
| Watermelon | *Kankana* |  |  |
| Guava | *Gwaba* |  | X |
| Sesame seeds | *Ridi* | X |  |
| Lettuce | *Salati* |  |  |
| Tomatoes | *Toumati* |  |  |

**Figure S1.** Pile Sort Results (t-SNE): Eating and Cooking Piles

**Figure S2.** Pile Sort Results (t-SNE): Health and Nutrition Piles

## Health and Nutrition Benefits of Foods

In pile sort exercises, respondents also grouped foods into categories based on their health properties. Table S2 shows the types of health and nutrition properties on which respondents based their pile creation and the common foods placed in each category. Respondents made the most piles for foods that are generally nutritious and food for the body, foods that increase blood and fight anemia, foods that give strength and build the body, and foods that have vitamins.

**Table S2.** Foods by Health and Nutritional Properties

| **Health or Nutrition Property** | **Food** |
| --- | --- |
| Increasing fat (*kara maski*) | **Legumes and seeds:** Tiger nut, groundnut |
| Fights hunger (*yana fidda gniwa*) | **Animal source foods:** *kilishi* (dried meat), eggs, liver, fish, meat  **Cereals:** Rice, wheat, sorghum, millet, guinea corn, sweet potatoes (vitamin A orange flesh)  **Legumes and seeds:** Sesame seeds, cowpea |
| Gives energy | **Animal source foods:** Liver, meat  **Cereals:** Rice, wheat  **Tubers:** Sweet potatoes (vitamin A orange flesh), taro, cassava |
| Gives strength (*karhi*) or builds the body | **Animal source foods:** Cheese, eggs, liver, locusts, milk  **Fruit:** Baobab fruit  **Vegetables:** Cabbage, moringa leaves |
| Good for growth (*kayan gina jiki*) | **Fruit:** Papaya, cantaloupe, watermelon, mango, guava, orange, banana, baobab fruit  **Legumes and seeds:** Soybean curds  **Tubers:** Yam  **Vegetables:** Wild eggplant, eggplant, carrots, pumpkin, dried tomatoes, cabbage, lettuce, onions, tomatoes |
| Has vitamins (*maganin gniwa*) | **Legumes and seeds:** Tiger nut, cowpea, groundnuts  **Vegetables:** Carrots, dried tomatoes |
| Improves vision | **Fruit:** Papaya, orange, cantaloupe, banana, watermelon, guava  **Tubers:** Taro, cassava, yam  **Vegetables:** Eggplant, wild eggplant, carrots, tomatoes, moringa leaves, sorrel, cabbage, amaranth leaves, pumpkin, onion, lettuce |
| Increases blood (*yana kara gini*) or fights against anemia (*kara jini*) | **Fruit:** Guava, papaya, watermelon, mango, orange, banana, cantaloupe  **Vegetables:** Wild eggplant, eggplant, carrots |
| Increases vigor (*kara karfin jiki*) or liveliness (*hamzari*) | **Animal source foods:** Milk, cheese  **Cereals:** Guinea corn  **Fruit:** Baobab fruit  **Legumes and seeds:** Cowpea  **Tubers:** Cassava, yam, sweet potatoes (vitamin A orange flesh), taro, potatoes |
| Nourishes skin (*yana djara fata*) | **Animal source foods:** Liver, *kilishi* (dried meat)  **Fruit:** Papaya, cantaloupe, guava, mango  **Legumes and seeds:** Tiger nut, sesame seeds, groundnuts  Oil  **Tubers:** Cassava, yam, sweet potatoes (vitamin A orange flesh)  **Vegetables:** Tomatoes, cabbage, amaranth leaves, pumpkin, lettuce |

## Food Access and Food Practices

Table S3 presents the foods respondents said are commonly consumed and available locally and the main sources for those foods, although these vary seasonally. We list foods in the row corresponding to the most common source. Within each category, the foods that respondents most frequently indicated their communities consume are in bold. With the exception of milk, animal source foods, including meat, eggs, fish, and cheese, were largely not accessible. Many respondents did not produce these foods and described them as expensive to purchase. Additional foods that respondents said were difficult to access included millet, sorghum, papaya, bananas, guava, carrots, eggplant, potatoes, and locusts. In the pile sort exercises, respondents most commonly labeled animal source foods (*kilishi* [dried meat], locusts, meat, liver, and eggs) and oil as luxury foods.

**Table S3.** Common Foods by Source and Food Group

| **Food Source** | **Cereals and Tubers** | **Legumes and Seeds** | **Animal Source Foods** | **Vegetables** | **Fruit** |
| --- | --- | --- | --- | --- | --- |
| Own production (farming or gardening) | **Millet**  **Sorghum**  **Cassava** | **Cowpeas**  **Groundnuts** | Not applicable (N/A) | **Baobab leaves**  **Sorrel**  **Squash**  **Okra**  Tomato | **Watermelon**  Melon |
| Purchase | Rice  Sweet potato | Soybeans | **Milk** | Cabbage  Lettuce | Orange |
| Gathered from the wild | N/A | N/A | N/A | *Laptadenia Hastata* (green leaves)  *Gui* (green Leaves) | N/A |
| Food distributions or vouchers | Millet  Flour  Rice | Beans | N/A | N/A | N/A |

We gathered information about how a range of foods are prepared and consumed through the pile sort activities and FGDs. These foods were not all accessible to respondents, however, so this information reflects local food norms and dishes, rather than the variety of what respondents eat day-to-day.

The main cereal staples consumed in Maradi and Zinder—millet and sorghum—are made into fufu (dough balls or paste resembling a stiff flour porridge), or traditional couscous. Additional ingredients in the dough balls may include soybeans, cheese, milk, sesame seeds, baobab fruit, guinea corn, cowpeas, and wheat. Some people consume maize dough, rice, or pasta as the starch in a meal, but more commonly, they consume millet and sorghum. Nigeriens eat the starch with sauces made with vegetables, legumes, tubers, or less frequently meat, for lunch, dinner, and sometimes breakfast. Adolescents also reported eating porridge, particularly for breakfast, and women discussed feeding children porridge. Fruits, depending on the season, were consumed on the side of main dishes.

Respondents described a range of ingredients that they use in sauces, provided the foods are accessible. The vegetables used in sauces are fresh or dried green leafy vegetables such as sorrel; baobab leaves; amaranth leaves; moringa leaves; and other vegetables like tomatoes, okra, or pumpkin. Legumes used in sauces include cowpeas, soybeans, and groundnuts. Tubers used in sauces include potatoes, taro, and cassava. They also add onion, oil, and other flavoring like Maggi cubes. Common sauces named by respondents include baobab leaf sauce, okra sauce, “red” sauce (i.e., tomato based), “black” sauce (with a base of dried leaves like baobab, or okra, which gives a sticky consistency). Ingredients for what respondents described as good leaf sauce (*kayan tabshe*) were groundnuts, sorrel, dried tomatoes, amaranth leaves, pumpkin, onion; they may also include onion, meat or fish, or other greens like moringa leaves or cabbage.

## References

1. Borgatti S. (2002) A statistical method for comparing aggregate data across a priori groups. *Field Methods* **14**, 88–107.
2. Borgatti S. (n.d.) Multidimensional Scaling. Analytic Technologies. http://www.analytictech.com/networks/mds.htm#:~:text=When%20the%20MDS%20map%20perfectly,or%20just%20%22Stress%201%22. (accessed June 2023).
3. van der Maaten L, & Hinton G. (2010) Visualizing non-metric similarities in multiple maps.” *Mach Learn* **87**, 33–55.
